# Cognitive Activity as a Moderator of Educational Attainment and Work Status in Cognitive Aging

**DOI:** 10.1093/geroni/igaa057.931

**Published:** 2020-12-16

**Authors:** Mirjam Stieger, Margie Lachman

**Affiliations:** Brandeis University, Waltham, Massachusetts, United States

## Abstract

Cross-sectional findings showed that education differences in memory performance were moderated by frequent cognitive activity (Lachman et al., 2010). The present study examined whether frequent cognitive activity could compensate for lower education when focusing on change in cognitive performance across nine years. The study also explored whether cognitive activity can slow down declines in retired adults as previous research suggested that retiring is associated with an increased risk of cognitive decline (e.g., Wickrama et al., 2013). Longitudinal data from the MIDUS study included N = 3,325 middle-aged and older adults. Outcome variables were two factors of cognitive performance: Episodic Memory (EM) and Executive Functioning (EF). Independent variables were years of education, work status (working vs. retired), and frequency of cognitive activity. The results suggest that cognitive activity moderated the effect of educational attainment on change in EM. Individuals with both higher education and cognitive activity showed the smallest declines in EM. Individuals with lower educational attainment but high cognitive activity had less decline in EM compared to their low education counterparts. Those who increased their cognitive activity over time showed less decline in EF. In terms of work status, working adults had less decline in EM and EF compared to retired adults and retired adults who did not maintain their cognitive activity declined more in EF. The results emphasize the importance of frequent engagement in cognitive activity across the lifespan, which can attenuate cognitive declines especially among those who have lower education or have retired.

